# Sequential EGFR T790M, MET c.3010C>G‐Associated Exon 14 Alteration, and RET‐CCDC6 Fusion in EGFR‐Mutant NSCLC: A Case Report

**DOI:** 10.1111/1759-7714.70322

**Published:** 2026-07-26

**Authors:** Yan Zhu, Chan Wang, Nan‐Lin Hu, Dan‐He Wang, Shi‐Kai Wu

**Affiliations:** ^1^ Department of Oncology Peking University First Hospital Beijing China

**Keywords:** acquired resistance, EGFR mutation, MET exon 14 alteration, non–small‐cell lung cancer, RET fusion

## Abstract

Acquired resistance to epidermal growth factor receptor (EGFR) tyrosine kinase inhibitors (TKIs) in EGFR‐mutant non–small‐cell lung cancer (NSCLC) is heterogeneous, and serial re‐biopsy may uncover actionable mechanisms. We report a 55‐year‐old man with stage IVB lung adenocarcinoma harboring EGFR L858R and TP53 R282W. First‐line icotinib produced a partial response (PR) with progression‐free survival (PFS) of 7 months. Rebiopsy at progression identified acquired EGFR T790M together with a MET c.3010C>G exon 14‐related alteration. Almonertinib induced a second PR lasting 7 months. After renewed progression, almonertinib plus savolitinib achieved another PR for 8 months, supporting MET‐dependent bypass resistance. Biopsy of a newly progressive iliac metastasis then showed emergence of RET‐CCDC6 fusion, whereas T790M and the MET alteration were no longer detected. Because selective RET inhibition was initially unaffordable, the patient received pemetrexed‐carboplatin plus continued almonertinib, followed by selpercatinib plus almonertinib. Local radiotherapy to oligoprogressive bone and lung lesions prolonged benefit from systemic therapy. At the latest follow‐up in January 2026, the patient remained alive nearly 50 months after diagnosis. This case illustrates clonal evolution under treatment pressure and highlights three practical lessons: repeat molecular testing at each clinically meaningful progression, matched combination targeted therapy for actionable bypass alterations, and local ablative radiotherapy for oligoprogression.

## Graphical abstract

Serial re‐biopsy in EGFR‐mutant NSCLC revealed sequential acquired EGFR T790M, MET c.3010C>G exon 14‐related alteration, and RET‐CCDC6 fusion. Mechanism‐guided therapy with almonertinib, almonertinib plus savolitinib, and later selpercatinib plus almonertinib with local radiotherapy achieved prolonged disease control, with survival approaching 50 months.

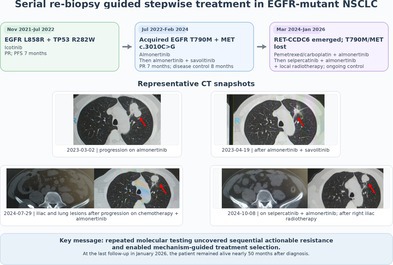

## 1 Introduction

Third‐generation growth factor receptor (EGFR) tyrosine kinase inhibitors (TKIs) are the preferred first‐line treatment for advanced non–small‐cell lung cancer (NSCLC) with common EGFR mutations, yet acquired resistance remains inevitable and molecularly heterogeneous [1, 2, 3, 4]. Off‐target resistance mechanisms account for roughly 20% of resistance in EGFR‐mutant NSCLC. Among these, aberrant MET signaling is a prominent pathway [5]. MET gene amplification—which drives HER3‐dependent activation of PI3K/AKT signaling—has been observed in ~5%–15% of patients progressing on almonertinib [5]. In rare cases, mutations affecting MET exon 14 splice‐site or exon 14‐related alterations may arise as acquired resistance to EGFR TKIs [6]. Another important bypass mechanism is the emergence of oncogenic fusions. RET fusions (such as CCDC6–RET or NCOA4–RET) can drive tumor growth independently of EGFR, and their appearance confers resistance to ongoing EGFR‐targeted treatment. Other bypass alterations (ALK fusions, HER2 amplifications, BRAF mutations, PIK3CA mutations, etc.) occur at lower frequencies, underscoring that EGFR‐mutant cancers can evolve through multiple pathways under treatment pressure [7].

We describe a patient with EGFR L858R‐mutant metastatic NSCLC who developed sequential EGFR T790M, MET exon 14‐related, and RET fusion‐mediated resistance and achieved prolonged survival through repeated biopsy, matched therapy, chemotherapy, and local radiotherapy.

## 2 Case Presentation

A 55‐year‐old Chinese man presented in November 2021 with progressive chest and upper back pain. Computed tomography, positron emission tomography/computed tomography, and abdominal magnetic resonance imaging showed a 5.7‐cm left upper lobe mass, mediastinal nodal disease, multiple bone metastases, and a small liver metastasis. Brain magnetic resonance imaging was negative. The clinical stage was cT3N3M1c (stage IVB). Computed tomography‐guided biopsy of the lung lesion confirmed poorly differentiated adenocarcinoma. Next‐generation sequencing (425‐gene panel) detected EGFR L858R and TP53 R282W. After discussion of guideline‐preferred first‐line third‐generation EGFR TKI, icotinib 125 mg twice daily was selected because of economic considerations. The best response was partial response (PR), and first‐line progression‐free survival (PFS) was 7 months (Table 1).

**TABLE 1:** Timeline of molecular evolution, treatment, and response.

Time	Clinical status	Molecular findings	Treatment	Outcome
Nov 2021	Diagnosis	EGFR L858R; TP53 R282W	Icotinib 125 mg twice daily	PR; PFS 7 months
Jul 2022	Progression in lung	Acquired EGFR T790M + MET c.3010C>G (exon 14‐related)	Brief icotinib escalation to 250 mg three times daily	No benefit; PD after ~6–7 weeks
Oct 2022‐May 2023	Post‐T790M treatment	No new biopsy during response	Almonertinib 110 mg once daily	PR; PFS 7 months
Jun 2023‐Feb 2024	Progression in lung/bone with persistent MET‐driven suspicion	No repeat biopsy; treatment selected based on previously detected MET c.3010C>G	Almonertinib + savolitinib 600 mg once daily	PR; disease control 8 months
Mar‐Oct 2024	New right iliac lesion	EGFR L858R retained; T790M and MET c.3010C>G lost; RET‐CCDC6 emerged	Pemetrexed (500 mg/m^2^) + carboplatin (AUC 5) every 3 weeks for 4 cycles + continued almonertinib	Stable disease; grade 3 neutropenia/thrombocytopenia
Oct 2024‐Jan 2026	Oligoprogression in iliac bone and lung	RET‐CCDC6 detected on prior iliac biopsy	Selpercatinib 160 mg twice daily + almonertinib; RT 30Gy in 10 fractions to iliac lesion and 50Gy in four fractions to lung lesion	Ongoing disease control at last follow‐up

Abbreviations: AUC, area under the curve; PD, progressive disease; PFS, progression‐free survival; PR, partial response; RT, radiotherapy.

**FIGURE 1:**
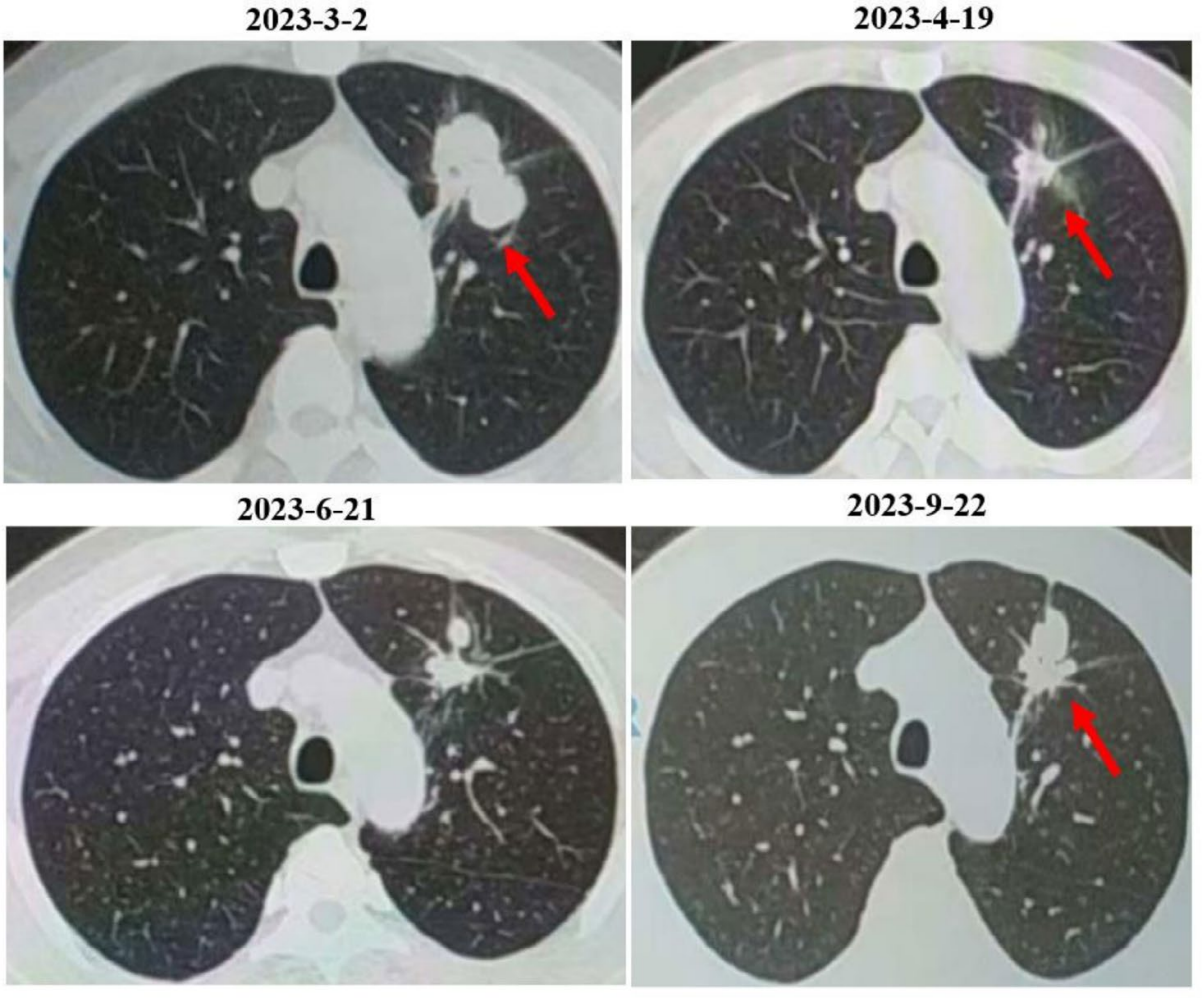
Selected axial chest CT images (lung window) showing interval changes in the primary lung lesion during late almonertinib monotherapy and subsequent almonertinib plus savolitinib treatment. The lesion (red arrows) initially decreased, then regrew at progression, and decreased again after addition of savolitinib.

At progression in July 2022, repeat biopsy of the lung lesion confirmed persistent EGFR L858R and newly acquired EGFR T790M together with MET c.3010C>G, annotated as an exon 14‐related alteration. While awaiting the molecular report, icotinib was briefly escalated to 250 mg three times daily, but disease progressed after approximately 6–7 weeks. Almonertinib 110 mg once daily was started in October 2022 and induced PR for 7 months. In May 2023, progression in the lung and bone occurred. Given the previously identified MET c.3010C>G exon 14‐related alteration and the clinical suspicion of MET‐dependent bypass resistance, almonertinib was combined with savolitinib 600 mg once daily in June 2023. This dual blockade again achieved PR, with disease control lasting 8 months (Figure 1, Table 1).

**FIGURE 2:**
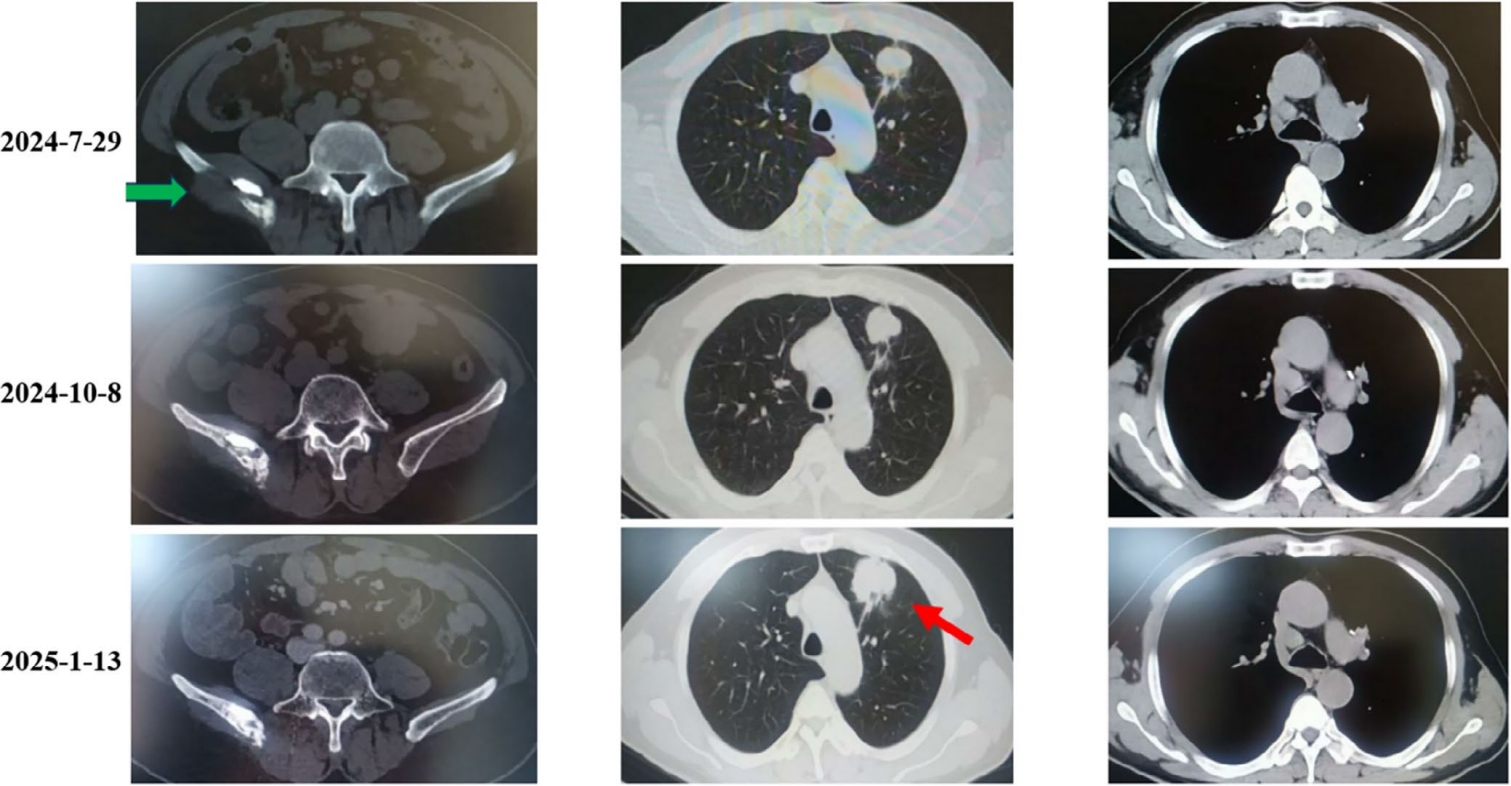
Axial CT images during sixth‐line dual targeted therapy with selpercatinib plus almonertinib combined with local radiotherapy. Left column: Axial pelvic CT showing the right iliac metastatic lesion (green arrow) at baseline (2024‐7‐29), interval progression before radiotherapy (2024‐10‐8), and postradiotherapy follow‐up (2025‐1‐13). Middle column: Axial chest CT (lung window) showing interval enlargement of the primary lung lesion (red arrow) before thoracic radiotherapy and postradiotherapy shrinkage on follow‐up (2025‐1‐13). Right column: Corresponding axial chest CT images in mediastinal window at the same time points.

After oligoprogression in early 2024, biopsy of a new right iliac metastasis showed persistent EGFR L858R, disappearance of T790M and MET c.3010C>G, and emergence of RET‐CCDC6 fusion. Because selective RET inhibition was initially unaffordable, pemetrexed 500 mg/m^2^ plus carboplatin [area under the curve (AUC) 5] every 3 weeks was administered for four cycles with continued almonertinib, achieving stable disease. Grade 3 neutropenia and thrombocytopenia required growth factor support and brief treatment delay. In October 2024, selpercatinib 160 mg twice daily was added to almonertinib. Oligoprogressive sites were treated with radiotherapy: 30Gy in 10 fractions to the right iliac lesion in November 2024 and 50Gy in four fractions to the primary lung lesion in December 2024. At the last follow‐up in January 2026, the patient remained alive nearly 50 months from diagnosis with ongoing disease control (Figure 2, Table 1).

## 3 Discussion

This case highlights the value of serial molecular reassessment in EGFR‐mutant NSCLC. Each clinically meaningful progression uncovered a new actionable mechanism and directly changed management: T790M supported almonertinib, the MET exon 14‐related alteration supported combined EGFR/MET inhibition, and RET‐CCDC6 supported combined EGFR/RET inhibition [8, 9]. The prolonged survival in this patient reflects adaptive treatment based on tumor evolution rather than empiric sequencing alone.

The MET codon 1004 alteration deserves particular comment. MET exon 14 encodes the juxtamembrane region containing Y1003, the CBL‐binding site that promotes receptor ubiquitination and degradation; alterations affecting this region can result in exon 14 skipping or functional loss of this negative regulatory motif, thereby enhancing downstream MET signaling [10]. In our patient, the assay annotated MET c.3010C>G as an exon 14‐related alteration, and the subsequent clinical response to almonertinib plus savolitinib supports its biologic relevance. However, no RNA assay or functional experiment was performed, so the mechanistic interpretation should be regarded as clinicogenomic rather than definitive. Additionally, beyond on‐target resistance, NSCLC progression is shaped by broader transcriptional and signaling plasticity [11].

This case also has practical implications for guideline‐based care. It does not by itself justify changing guidelines, but it reinforces existing recommendations to use a first‐line third‐generation EGFR TKI whenever feasible, to repeat molecular testing at progression, and to consider matched combination therapy for actionable bypass resistance [12]. In addition, local ablative radiotherapy helped control oligoprogressive lesions and prolong benefit from systemic therapy [13].

## Author Contributions

**Yan Zhu:** data curation, writing – original draft, investigation. **Dan‐He Wang:** data curation, writing – review and editing. **Nan‐Lin Hu:** writing – review and editing, data curation. **Chan Wang:** data curation, writing – review and editing. **Shi‐Kai Wu:** supervision, writing – review and editing, methodology.

## Funding

The authors have nothing to report.

## Ethics Statement

The authors have nothing to report.

## Consent

The patient provided consent for publication of the clinical data and images.

## Conflicts of Interest

The authors declare no conflicts of interest.

## Data Availability

All relevant data are included in this article.
